# A systematic review of studies using network analysis to assess dynamics of psychotic-like experiences in community samples

**DOI:** 10.1017/S0033291725000261

**Published:** 2025-02-19

**Authors:** Błażej Misiak, Aleksandra Pytel, Bartłomiej Stańczykiewicz

**Affiliations:** 1 Department of Psychiatry, Wroclaw Medical University, 50-367 Wroclaw, Poland; 2 Division of Internal Medicine Nursing, Department of Nursing and Obstetrics, Faculty of Health Science, Wroclaw Medical University, 51-618 Wroclaw, Poland; 3 Division of Consultation Psychiatry and Neuroscience, Department of Psychiatry, Wroclaw Medical University, 50-367 Wroclaw, Poland

**Keywords:** comorbidity, mental disorder, network analysis, psychosis, psychotic symptoms, stress

## Abstract

Several studies have used a network analysis to recognize the dynamics and determinants of psychotic-like experiences (PLEs) in community samples. Their synthesis has not been provided so far. A systematic review of studies using a network analysis to assess the dynamics of PLEs in community samples was performed. Altogether, 27 studies were included. The overall percentage ranks of centrality metrics for PLEs were 23.5% for strength (20 studies), 26.0% for betweenness (5 studies), 29.7% for closeness (6 studies), 26.9% for expected influence (7 studies), and 29.1% for bridge expected influence (3 studies). Included studies covered three topics: phenomenology of PLEs and associated symptom domains (14 studies), exposure to stress and PLEs (7 studies), and PLEs with respect to suicide-related outcomes (6 studies). Several other symptom domains were directly connected to PLEs. A total of 6 studies investigated PLEs with respect to childhood trauma (CT) history. These studies demonstrated that PLEs are directly connected to CT history (4 studies) or a cumulative measure of environmental exposures (1 study). Moreover, CT was found to moderate the association of PLEs with other symptom domains (1 study). Two studies that revealed direct connections of CT with PLEs also found potential mediating effects of cognitive biases and general psychopathology. PLEs were also directly connected to suicide-related outcomes across all studies included within this topic. The findings imply that PLEs are transdiagnostic phenomena that do not represent the most central domain of psychopathology in community samples. Their occurrence might be associated with CT and suicide risk.

## Introduction

Although great progress has been made to improve the reliability of psychiatric diagnosis is apparent across subsequent editions of international diagnostic systems, the validity of psychiatric diagnosis remains problematic (Jablensky 2016). Several psychiatric disorders represent heterogeneous constructs with a variety of symptomatic manifestations, contributing risk factors, potential underlying mechanisms, and outcomes (Feczko et al. 2019). Some authors posit that discrete diagnostic entities with sufficient validity are not possible to be dissected (Kendell and Jablensky 2003). Consequently, it has been suggested that the operationalization of clinical manifestations within domains of psychopathology might be an alternative to traditional categorical approaches.

These considerations might be of particular relevance to psychotic disorders (Misiak et al. 2023a; Valle 2022). It has been proposed that the psychosis dimension forms a continuum between clinical and non-clinical populations (Stefanis et al. 2002). Indeed, it has been shown that some individuals from the general population report subclinical phenomena that resemble hallucinations and delusions but cannot be the basis to diagnose psychotic disorders because of their low impact on social functioning and low level of associated distress. These phenomena are commonly referred to as psychotic-like experiences (PLEs) (Kelleher and Cannon 2011; Hinterbuchinger and Mossaheb 2021; van Os et al. 2009). Studies based on taxometric approaches have also shown that PLEs and psychotic symptoms are better conceptualized as dimensional phenomena rather than taxonic (categorical) constructs in community samples (Adjorlolo et al. 2021; Taylor et al. 2016), individuals at clinical high risk of psychosis (Elahi et al. 2017), and patients with schizophrenia (Cuesta et al. 2007). Epidemiological studies have estimated the mean lifetime prevalence of PLEs at almost 6% (McGrath et al. 2015). In about one-third of affected individuals, PLEs are known to persist each year (Staines et al. 2023).

Although PLEs generally represent subclinical phenomena, their presence might have a clinical relevance. It has been found that the presence of PLEs might be related to a fourfold higher risk of psychosis and a threefold higher risk of mental disorder (Healy et al. 2019). These observations suggest that PLEs are not specifically related to the risk of psychosis spectrum disorders but might represent the phenomena indicating a broader risk of mental disorders. A greater utilization of mental health services among people with PLEs has also been observed indicating that in some individuals they might serve as the source of distress (Bhavsar et al. 2018). Moreover, a previous meta-analysis revealed that the presence of PLEs predicts the occurrence of suicidal ideation, suicide attempts, and suicide death (Yates et al. 2019). The authors of this meta-analysis found that the observations for suicidal ideation and suicide attempts cannot be explained by co-occurring psychopathology. There is also evidence that PLEs are bidirectionally associated with the occurrence of non-suicidal self-injury (NSSI) (Steenkamp et al. 2023; Zhou et al. 2024).

Several factors are known to increase the risk of PLEs, including pre- and perinatal complications, infections during a developmental period, altered neuroanatomical development, a higher urbanicity, ethnic minority status, low socioeconomic status, a history of traumatic events, tobacco, and cannabis use (Staines et al. 2022). These factors largely overlap with those found to increase the risk of psychosis. Similar psychological processes have also been demonstrated to play a role in the development of PLEs. For instance, it has been shown that PLEs are associated with systemic errors in cognitive processing and content, commonly referred to as cognitive biases (Gaweda et al. 2024; Livet et al. 2020). These include, that is jumping to conclusions, attention to threat, aberrant salience, externalizing bias, and belief inflexibility (Livet et al. 2020). Moreover, cognitive biases might mediate the association of childhood trauma with PLEs and psychosis (for review see Gaweda et al. (2024)).

The complexity of risk factors and potential outcomes of PLEs suggests the necessity to use comprehensive analytical models to better understand these phenomena. Network analysis is an approach that allows one to address multiple symptoms, behaviors, and psychological mechanisms in a single model. It is based on the assumption that psychological phenomena are dynamic and need to be analyzed in a full spectrum without imposing specific causal inferences. Moreover, it allows to indication of critical variables in the model, providing grounds to develop interventions focused on specific and well-defined targets. In recent years, there has been an exponential increase in the use of network analysis models of psychopathology (Robinaugh et al. 2020). This increase is also apparent across studies that aim to address various aspects related to the occurrence of PLEs in the general population. However, a synthesis of data from these studies has not been provided so far. In this regard, the present study aimed to provide a systematic review of studies investigating PLEs in community samples using a network analysis.

## Methods

### Protocol and reporting guidelines

The present systematic review was performed in agreement with the Preferred Reporting Items for Systematic Reviews and Meta-Analyses (PRISMA) 2020 Statement (Page et al. 2021). The protocol can be found in the Open Science Framework (OSF) registries (doi: 10.17605/OSF.IO/ZBKTV).

### Search strategy

Two reviewers (B.S. and A.P.) carried out independent online searches using the following combination of keywords: “psychotic” OR “delusion” OR “hallucination” AND “network analysis” OR “network perspective.” Online searches covered publication records from 6 databases including the APA PsycArticles, the Academic Search Ultimate, the ERIC, the Health Source: Nursing/Academic Edition, the MEDLINE Ultimate (including PubMed), and the CINAHL Ultimate. Online searches covered the period until 22 March, 2024 and no time restrictions were applied. All discrepancies about the inclusion of specific publication records were resolved through discussion with the third reviewer (B.M.).

### Eligibility criteria

Specific studies were included if they met all of the following criteria: (1) studies using a quantitative assessment of PLEs with self-reports and/or structured in-person interviews, (2) cross-sectional or longitudinal studies, (3) studies performed in community samples, and (4) studies based on network analysis. The following types of records were excluded: (1) observational studies based on samples of individuals with established diagnoses of mental disorder, (2) non-original articles (e.g. reviews, editorials, and commentaries), (3) unpublished manuscripts, (4) conference abstracts, (5) studies based on the analysis of social networks, (6) studies based on the analysis of brain networks, (7) case reports, and (8) non-English language publications.

### Data extraction

A data extraction template was used to collect the general characteristics of included studies: (1) age (mean ± SD), (2) gender, (3) study design (cross-sectional vs. longitudinal), (4) the tool used to assess PLEs, and (5) constructs assessed using network analysis (other than PLEs). Next, information about various aspects of a network analysis was extracted:

1. *The number of nodes:* The network analysis shows the main results as nodes that are connected with edges (Epskamp et al. 2018a). Nodes refer to specific variables included in the network. In turn, edges reflect the weights of connections between nodes. Thicker edges correspond with greater weights of visualized connections. Positive and negative associations are usually shown with different colors.
2. *Network estimation methods:* Gaussian graphical models (GGM) are used to assess normally distributed continuous variables (Epskamp et al. 2018b). Association networks and Ising models have been developed for binary data (Haslbeck et al. 2022). Mixed graphical models (MGM) are for networks composed of continuous and binary data (Haslbeck and Waldorp 2020).
3. *Centrality metrics:* The importance of specific nodes in the network is shown by calculating their centrality (Bringmann et al. 2019). There are four centrality metrics used in network analysis: strength, betweenness, closeness, and expected influence. Strength centrality shows the sum of edge weights connected to a specific node. Betweenness illustrates how many times a specific node is located on the shortest pathways between two other nodes. Closeness is the inversed total length of shortest pathways between a specific node and all other nodes in the network. Similar to strength, expected influence is also the sum of edge weights but takes into consideration the presence of negative edges (Robinaugh et al. 2016). In some studies, bridge centrality metrics are assessed (Jones et al. 2021). They represent the centrality of specific nodes with respect to the nodes from all other communities in the network. Centrality metrics are shown as the order of importance of nodes in the network.
4. *Node predictability:* Predictability refers to the percentage of variance explained by nodes directly connected to a specific node (Haslbeck and Fried 2017). It is often visualized as a filled part of the ring around each node. Predictability is usually strongly correlated with centrality metrics. As opposed to centrality metrics, predictability is reported using absolute values.
5. *Assessment of network stability:* Stability can be assessed by calculating the correlation stability coefficient (CS-C). The CS-C value is the proportion of the sample that can be dropped while maintaining the correlation strength of at least 0.70 with the original centrality score and edge weights. The network analysis is considered stable when the CS-C value is at least 0.25 (ideally it should be higher than 0.50) (Epskamp et al. 2018a).
6. *Assessment of network accuracy:* Bootstrapped 95% confidence intervals provide information about the accuracy of edges (Epskamp et al. 2018a). Moreover, bootstrapping procedures allow to indication of significant differences between specific edge weights.
7. *The comparison of networks:* Two networks can be compared with respect to the global strength showing the overall strength of node connectivity. Additionally, edge weights can be compared across two networks. These analyses can be carried out using the network comparison test (NCT), that is a resampling-based permutation test (van Borkulo et al. 2022).
8. *Reproducibility:* For each study, we recorded if the code and dataset used for a network analysis were available.

### Data synthesis

A qualitative data synthesis was carried out. First, the general characteristics of eligible studies and their quality were discussed. Quality assessment was performed using the tool developed for cohort and cross-sectional studies by the US National Heart, Lung, and Blood Institute (https://www.nhlbi.nih.gov/health-topics/study-quality-assessment-tools). This tool is based on 14 items recording various aspects of study quality using yes-or-no responses. The total quality score ranges between 0 and 14. Quality can be rated as poor (a score of 0–4), fair (a score of 5–10), and good (a score of 11–14). Next, various methodological aspects of a network analysis were synthesized. To synthesize the findings with respect to the centrality of PLEs, the highest percentage rank of centrality metrics for PLEs was estimated by dividing reported centrality by the number of nodes in the network. The overall percentage centrality rank was estimated by calculating mean values across all included studies. However, studies that limited a network analysis to the nodes representing PLEs and those that included only one node representing variables other than PLEs were excluded from this analysis. Finally, the main findings were described across specific topics identified after reviewing eligible publication records.

## Results

### The general characteristics of eligible studies

**Figure 1.**
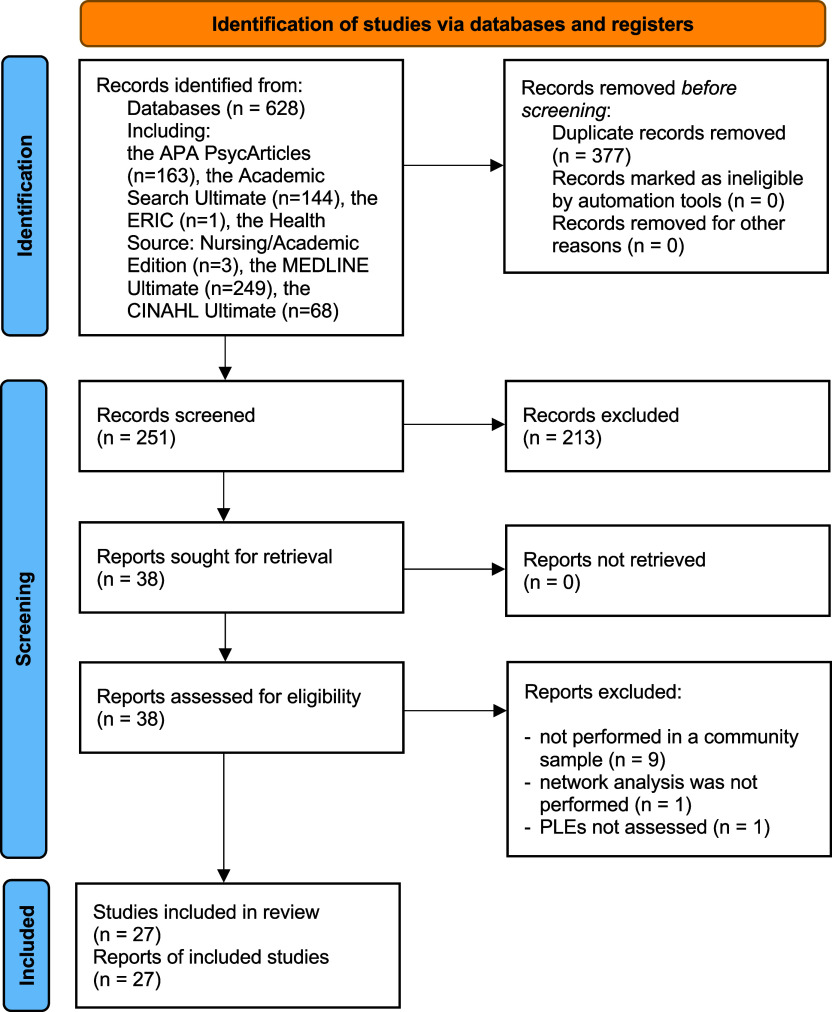
The PRISMA flow diagram.

Out of 628 publication records identified, a total of 27 studies were found eligible for a systematic review (Figure 1, Table 1). The majority of studies (*N* = 24) limited a network analysis to cross-sectional data. Only six studies included samples of children and/or adolescents (Cheng et al. 2024; Fonseca-Pedrero et al. 2021; Nunez et al. 2018; Nunez et al. 2020; Qiao et al. 2024; Sun and Zhong 2023). The quality of studies ranged between 4 and 11. The majority of studies had the quality rated as fair (*N* = 25). Other studies showed poor (*N* = 1) and good quality (*N* = 1).

**Table 1.** Summary of studies included in a systematic review

Study	Study characteristics				Measures		Network analysis					Reproducibility	Quality
	*N*	Study design^a^	Age^b^	Gender (%F)	The scale for PLEs	Other constructs assessed using a network analysis	*N* ^c^	Centrality and predictability	Model	CS-C	Edge accuracy		
Astill Wright et al. (2023)	4472	Cross-sectional	23–24	63.3	PLIKSi	Negative symptoms, depressive symptoms, anxiety symptoms, PTSD symptoms, trauma exposure	45	Strength, Bridge EI, Bridge Strength, Bridge Clo., Bridge Bet.	GGM	–	95% CI, Diff. Test	–	6
Betz et al. (2023)	7242	Cross-sectional	50.0 (44.1)	56.8	PSQ	Mood symptoms, domestic violence, physical abuse, sexual abuse, cannabis use, ethnicity, sex	6	–	GGM	0.28–0.75	95% CI^d^	Dataset, code	8
Cernis et al. (2021)	6941	Cross-sectional	40.3 (15.7)	87.2	SPEQ	Felt sense of anomaly, anxiety, depression, insomnia, PTSD symptoms, distress tolerance	11	Strength, Clo., Bet.	GGM	0.75	95% CI, Diff. Test	Code	5
Cheng et al. (2024)	5008	Cross-sectional	12.9 (1.3)	51.0	CAPE–42	–	42	EI, bridge EI, Pred.	GGM	0.75	95% CI, Diff. Test	–	6
Deng et al. (2023)	247	Cross-sectional	37.8 (10.8)	52.6	R-GPTS	Depression, social anxiety, social functioning, COVID–19-related preoccupation, interpretation inflexibility	7	–	GGM, MGM	–	–	–	5
Fonseca-Pedrero et al. (2021)	1790	Cross-sectional	15.7 (1.3)	53.7	PQ-B	Suicidality, general psychopathology, quality of life, depression, self-esteem, dishonesty	11 and 21	EI, Pred.	Ising, GGM	–	95% CI	–	5
Fung et al. (2024)	468	Cross-sectional	25.6 (8.6)	91.0	CAPE–42	Dissociation, PTSD symptoms, traumatic events	18	Strength, Clo., Bet.	GGM	0.75	95% CI	–	7
Gaweda et al. (2021)	6772	Cross-sectional	26.5 (4.7)	63.3	PQ–16	Depression, cognitive biases, childhood trauma	34	Strength, Pred.	MGM	0.75	95% CI, Diff. Test	Code	6
Hajduk et al. (2023)	649	Cross-sectional	40.2 (13.1)	51.3	CAPE–42	Autistic traits, social relationships	21	Strength, EI	GGM	0.595, 0.672	95% CI	–	4
Huang et al. (2023)	1813, 427	Cross-sectional	22.7 (3.5), 21.8 (1.1)	78.0, 79.2	MSS	Depression, motivation to reward, bipolar disorder traits, childhood trauma	14	Strength, Clo., Bet., EI, Pred.	GGM	0.75, 0.67	95% CI, Diff. Test	–	6
Misiak et al. (2023b)	1647	Cross-sectional	25.8 (4.9)	88.0	PQ–16	Narcissism, cognitive biases, metacognition, emotion regulation, age, gender, education, lifetime history of psychiatric treatment	18	Strength, Pred.	MGM	0.75	95% CI, Diff. Test	–	6
Misiak et al. (2023c)	4203	Cross-sectional	25.3 (5.7)	63.8	PQ–16	Depressive symptoms, NSSI, childhood trauma	35	Strength, Pred.	MGM	0.75	95% CI, Diff. Test	Dataset	7
Misiak and Frydecka (2024)	581	Longitudinal	27.9 (5.0)	49.2	PQ–16	Intent to seek treatment	15	Strength, Pred.	MGM	0.36	95% CI, Diff. Test	–	10
Misiak et al. (2024)	4203	Cross-sectional	25.3 (5.7)	63.8	PQ–16	Depressive symptoms, insomnia, suicidal ideation, age, gender, education, occupation	29	Strength	MGM	0.59, 0.36	95% CI, Diff. Test	Dataset	6
Murphy et al. (2018)	34653	Cross-sectional	N/P	N/P	AUDADIS-IV	–	16	Strength, Clo., Bet.	GGM, Ising	0.44, 0.59, 0.75	95% CI, Diff. Test	Code	6
Nunez et al. (2018)	1685	Cross-sectional	16.0 (1.5)	54.1	CAPE-P15	Social anxiety, negative symptoms, suicidal ideation	15	Strength, Bet.	Ising	0.13, 0.21, 0.28	95% CI, Diff. Test	–	6
Nunez et al. (2020)	1591	Cross-sectional	16.0 (1.5)	53.4	CAPE-P15	Social anxiety, negative symptoms, suicidal ideation	22	Strength	Ising	0.60	95% CI, Diff. Test	–	6
Qiao et al. (2024)	865	Cross-sectional and longitudinal	12–20	67.0	PQ–16, DISC-C	Stress, negative affect, loneliness, threat anticipation, general psychopathology, attachment insecurity, maladaptive cognitive emotion regulation	24, 26	Strength, Clo., Bet., EI, Bridge Strength	GGM	0.75	95% CI, Diff. Test	–	8
Rejek and Misiak (2023)	1100	Cross-sectional	26.3 (5.2)	51.4	PQ–16	Depressive symptoms, manic symptoms, ADHD symptoms, OCD symptoms, anxiety symptoms	11	Strength, Pred.	MGM	0.36, 0.59	95% CI, Diff. Test	–	6
Rejek and Misiak (2024)	1100	Cross-sectional	26.3 (5.2)	51.4	PQ–16	Exposome score	17	Strength, Pred.	MGM	0.44	95% CI, Diff. Test	–	6
Scott et al. (2021)	1867	Cross-sectional	26.4 (7.5)	57.0	BMC Psychosis Assessment	Depressive and hypomanic symptoms	20	Strength, Clo., Bet.	Ising	–	95% CI	–	6
Suen et al. (2024)	2186	Longitudinal	19.8 (2.8)	58.0	PQ-B	ADHD symptoms, ASD symptoms, depressive symptoms, anxiety symptoms, social functioning, health-related quality of life	21	EI, Bridge EI	GGM	–	95% CI	Code	11
Sun and Zhong (2023)	1199	Cross-sectional	15.9 (1.0)	56.0	CAPS	Bullying	23, 27, 32	EI	GGM	0.28, 0.36	95% CI	–	6
Wusten et al. (2018)	7141	Cross-sectional	27.4 (10.0)	48.6	CAPE–42	–	20	Strength, Bet., Clo.	GGM	0.34–0.75	–	Dataset, code	7
Xavier et al. (2022)	5094	Cross-sectional	11–17	52.0	PS-R, SOPS	Other domains of psychopathology	14	Strength	GGM	–	–	Code	7
Yang et al. (2023)	4761	Cross-sectional	18.6 (1.0)	55.5	CAPE-P15	Depressive symptoms, manic symptoms, anxiety symptoms	6, 44	Strength	GGM, MGM	0.75, 0.67	95% CI	–	5
Zhou et al. (2023)	2328	Cross-sectional	18.4 (0.7)	72.9	CAPE–8	Suicidality, NSSI, depressive symptoms, internet addiction, childhood trauma, cyberbullying, social support, family-related factors, alcohol use, cigarette smoking	22	Strength, EI	MGM	0.75	95% CI	–	5

*Note:* ADHD, attention-deficit/hyperactivity disorder; AUDADIS-IV, the Alcohol Use Disorder and Associated Disabilities Interview Schedule-IV (Grant et al. 2003); Bet., betweenness; BMC Psychosis Assessment, the Brain and Mind Centre Psychosis Assessment (Yung et al. 2009), CAPE, the Community Assessment of Psychic Experiences (Stefanis et al. 2002); CAPS, the Cardiff Anomalous Perceptions Scale (Bell et al. 2006); CS-C, correlation stability coefficient; Clo., closeness; Diff. Test, bootstrapped edges comparison; DISC-C, the Diagnostic Interview Schedule for Children (Costello et al. 1985); EI, expected influence; GGM, Gaussian Graphical Model; MGM, Mixed Graphical Model; MSS, the Multidimensional Schizotypy Scale (Kwapil et al. 2018); N/P, not provided; NSSI, non-suicidal self-injury; OCD, obsessive-compulsive disorder; PLEs, psychotic-like experiences; PLIKSi, the Psychosis-Like Symptoms Semi-structured Interview (Sullivan et al. 2020); PQ-16, the Prodromal Questionnaire-16 (Ising et al. 2012); PQ-B, the Psychosis Questionnaire Brief (Loewy et al. 2011); PS-R, the PRIME Screen – Revised (Kobayashi et al. 2008); PSQ, the Psychosis Screening Questionnaire (Bebbington and Nayani 1995); PTSD, post-traumatic stress disorder; R-GPTS, the Revised Green et al. Paranoid Thoughts Scale (Freeman et al. 2021); SOPS, the Scale of Prodromal Syndromes (Miller et al. 2003); SPEQ, Specific Psychotic Experiences Questionnaire (Ronald et al. 2014)

a Refers to the use of a network analysis

b Data expressed as mean (SD), range, or specific age of participants

c *N* refers to the number of network nodes

d Refers to the analysis of 95% confidence intervals for edge-weight accuracy

In the majority of studies, self-reported measures of PLEs were used. The Prodromal Questionnaire-16 (Ising et al. 2012) and the Community Assessment of Psychic Experiences (Stefanis et al. 2002) were the most commonly used questionnaires (*N* = 8 for both measures). Only four studies used in-person assessment of PLEs with structured interviews in the whole sample or a part of the sample (Astill Wright et al. 2023; Murphy et al. 2018; Qiao et al. 2024; Xavier et al. 2022).

### Network characteristics

The number of nodes included in the network varied between 6 (Betz et al. 2023; Yang et al. 2023) and 45 (Astill Wright et al. 2023). In the majority of network models, only continuous variables were analyzed using GGM. The network comparison tests were applied by four studies (Cheng et al. 2024; Huang et al. 2023; Scott et al. 2021; Wusten et al. 2018; Xavier et al. 2022). In one study, the recursive partitioning approach (Jones et al. 2020) was used to test several moderators (Betz et al. 2023).

**Figure 2.**
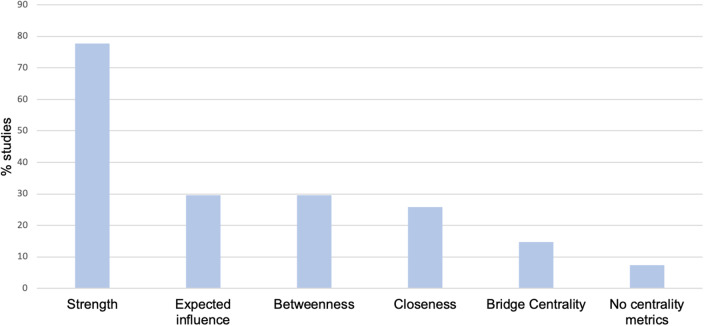
The percentage of studies using specific centrality metrics.

Node centrality was assessed using various metrics; however, the strength centrality was most frequently analyzed (*N* = 21, 77.8%) while any measures of the bridge centrality were least frequently used (*N* = 4, 14.8%, Figure 2). The overall percentage ranks of centrality metrics for PLEs were 23.5% for strength (20 studies), 26.0% for betweenness (5 studies), 29.7% for closeness (6 studies), 26.9% for expected influence (7 studies), and 29.1% for bridge expected influence (3 studies) (Supplementary Table 1). Only nine studies (33.3%) assessed node predictability.

The CS-C value was reported by 21 studies (77.8%). Only one study (Nunez et al. 2018) revealed insufficient network stability (CS-C < 0.25). Any measures of edge accuracy were reported by the majority of studies (*N* = 24, 88.9%). Reproducibility of data analysis was not commonly ensured. The code for data analysis was provided by six studies (22.2%). In turn, access to analyzed datasets was provided by four studies (14.8%).

### Topics addressed by network analysis studies

A detailed summary of the main findings across specific studies is shown in Supplementary Table 2. The analysis of reported findings revealed three distinct topics addressed by eligible studies: (1) phenomenology of PLEs and associated symptom domains, (2) exposure to stress and PLEs, and (3) PLEs and suicide-related outcomes.

*Phenomenology of PLEs and associated symptom domains:* A total of 14 studies (51.9%) focused on the analysis of the phenomenology of PLEs and associated psychopathological domains. Included studies revealed that PLEs show transdiagnostic connections with symptoms of autism spectrum disorder (ASD), attention-deficit/hyperactivity disorder (ADHD), anxiety, dissociation (especially identity dissociation), depression, mania, obsessive–compulsive disorder (OCD), post-traumatic stress disorder (PTSD), and narcissistic grandiosity (Cheng et al. 2024; Rejek and Misiak 2023; Misiak et al. 2023b; Yang et al. 2023). However, single studies reported that OCD symptoms (Rejek and Misiak, 2023) and specific depressive symptoms (i.e. “failure,” “guilty,” and “no future”) (Cheng et al. 2024) might be more strongly associated with PLEs. Also, with respect to specific PLEs, it has been demonstrated that persecutory ideation might be most strongly associated with depressive and anxiety symptoms, while bizarre experiences might be most strongly related to manic symptoms (Yang et al. 2023). However, the study by Scott et al. (2021) did not demonstrate that PLEs might be helpful in diagnosing bipolar disorder or differentiating individuals at high familial risk of bipolar disorder from unipolar depression cases. Compared with other domains of psychopathology, it is also important to note that PLEs have been found to show the lowest concordance of reporting by adolescents and their caregivers (Xavier et al. 2022).

Three studies focused on exploring PLEs with respect to associated distress (Murphy et al. 2018; Wusten et al. 2018) and help-seeking (Misiak & Frydecka 2024). The comparison of the presence and impairment/distress network of PLEs revealed a similar structure; however, the impairment network appeared to show a stronger interconnectivity (Murphy et al. 2018). Stronger interconnectivity of PLEs together with higher levels of associated distress have also been found among individuals from high-income countries compared with those representing low- and middle-income countries (Wusten et al. 2018). It has been suggested that more densely connected networks might reflect a greater likelihood of activation proneness between specific nodes corresponding with a higher level of vulnerability (van Borkulo et al. 2015). At least theoretically, the distress or impairment related to PLEs, might make individuals likely to seek help. One study included in this systematic review demonstrated that a self-reported presence of PLEs might predict the perceived need to seek help (Misiak & Frydecka 2024). This observation was reported for “deja vu experiences,” “problems in differentiating reality and imagination,” “a lack of control over own ideas or thoughts,” “being distracted by distant sounds,” and “paranoid thoughts.”

*Exposure to stress and PLEs:* The role of stress in the development of PLEs was addressed by 7 studies (26.9%). A total of 6 studies focused on the role of childhood trauma history. Among them, 4 studies revealed direct connections between childhood trauma history with PLEs (Gaweda et al. 2021; Huang et al. 2023; Qiao et al. 2024; Sun and Zhong 2023), 1 study tested moderating effects of childhood history in the association of PLEs with other domains of psychopathology (Betz et al. 2023), and 1 study included a history of childhood trauma in a composite measure of environmental exposures (Rejek and Misiak 2024). Two studies that revealed direct connections of a childhood trauma history with PLEs also found potential mediating effects of other processes including cognitive biases (Gaweda et al. 2021) and general psychopathology (Qiao et al. 2024). However, included studies revealed that childhood trauma history might also be related to other symptom domains including depressive and anxiety symptoms (Huang et al. 2023; Rejek and Misiak 2024).

An interesting approach to performing network analysis was adopted by Betz et al. (2023). The authors used a novel recursive partitioning approach to model the moderating effects of several variables (i.e. age, gender, ethnicity, deprivation, childhood abuse, separation from parents, bullying, domestic violence, cannabis use, and alcohol use) on the associations between depressive and anxiety symptoms, PLEs, and sleep disturbance. Heterogeneity across network dynamics appeared to be largely explained by gender. The authors found that a history of childhood abuse was associated with stronger connections between anxiety and PLEs in women.

Finally, one study investigated PLEs with respect to preoccupation related to the COVID-19 pandemic (Deng et al. 2023). In this study, interpretation inflexibility was associated with social functioning impairment. Affective symptoms and paranoia mediated this association. These associations were also magnified by stress experienced during the COVID-19 pandemic, that is a moderated mediation was found only in relation to affective symptoms, but not paranoia. A network analysis confirmed the moderating effects of the COVID-19-related preoccupation on the association between interpretation inflexibility and depression.

*PLEs and suicide-related outcomes:* Suicide-related outcomes were assessed using network analysis with respect to PLEs by 6 studies (23.1%). In all of these studies, PLEs were directly connected to suicide-related outcomes. Among them, 5 studies revealed that PLEs, especially perceptual anomalies and bizarre experiences, are directly connected to suicidal ideation and/or behaviors (Fonseca-Pedrero et al. 2021; Misiak et al. 2023c; Misiak et al. 2024; Nunez et al. 2018; Nunez et al. 2020). One study revealed that PLEs are connected to NSSI, suicidal ideation, and behaviors through the bridging effect of depressive symptoms (Zhou et al. 2023). Importantly, another study demonstrated that PLEs are directly connected to suicidal ideation only in participants with higher levels of insomnia (Misiak et al. 2024). In this study, the nodes representing PLEs that were directly connected to suicidal ideation included deja vu experiences, auditory hallucination-like experiences, and paranoia.

## Discussion

### Main findings

**Figure 3.**
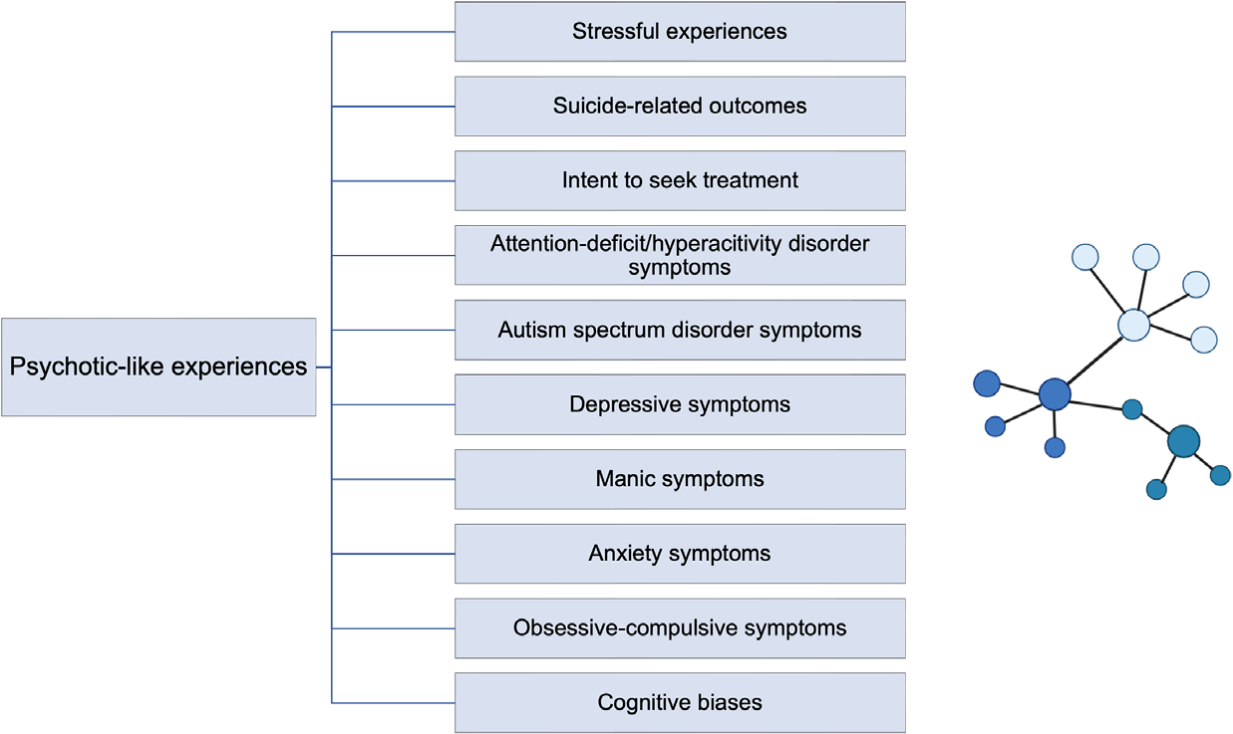
Overview of main findings of a systematic review. Psychotic-like experiences (PLEs) represent transdiagnostic markers of mental disorders (i.e. their occurrence is associated with various domains of psychopathology). In some cases, PLEs are the source of distress, contribute to help-seeking behaviors, and might be related to increased suicide risk and the occurrence of non-suicidal self-injury (NSSI). Stressful experiences, especially a history of childhood trauma, can influence the occurrence of PLEs through the effects on other domains of psychopathology and cognitive biases. Temporal ordering and/or causal associations cannot be concluded because of evidence from cross-sectional studies.

A brief overview of the main findings is shown in Figure 3. In general, findings from the present systematic review indicate that PLEs serve as a transdiagnostic phenomenon that might occur in the context of various mental disorders. However, there is some evidence that OCD symptoms might be more closely related to PLEs compared with other dimensions of psychopathology (Rejek and Misiak 2023). Indeed, the majority of included studies revealed that PLEs are not ranked among the most central nodes in the network. Altogether, these findings are in agreement with other studies showing that PLEs might predict the occurrence of mental disorders that are not limited to the psychosis spectrum (Giocondo et al. 2021; Lindgren et al. 2022). However, still little is known about the association between personality traits and PLEs. Only one study included in this systematic review revealed that narcissistic grandiosity might make individuals more prone to develop PLEs (Misiak et al. 2023b). This process appeared to be mediated by external attribution biases, the need to control thoughts, and emotion regulation through fantasizing.

Although PLEs are generally perceived as transdiagnostic phenomena, our analysis did not demonstrate that they are the most central network nodes. Indeed, the centrality percentage rank for PLEs varied between 23.5 for strength and 29.1 for bridge expected influence. These counterintuitive findings might be explained by the observations that other symptom domains and psychological processes have been found to show stronger connections. For instance, one study included in the present systematic review demonstrated that while PLEs are connected to all symptom domains assessed, depressive symptoms had a higher centrality rank than PLEs (Rejek and Misiak, 2023). Indeed, depressive symptoms may occur across various mental disorders and are also among the most commonly assessed psychopathological dimensions with respect to transdiagnostic associations (Fusar-Poli et al. 2019). However, it remains needed to further examine whether specific symptom dimensions differ in terms of their transdiagnostic dimensionality.

Although PLEs are known to serve as subclinical phenomena, they might be the source of distress and impairment in some individuals. In one of the included studies, PLEs were found to predict the perceived intent to seek treatment after 6 months (Misiak & Frydecka 2024). This observation was found for “déjà vu experiences,” “problems in differentiating reality and imagination,” “a lack of control over own ideas or thoughts,” “being distracted by distant sounds,” and “paranoid thoughts.” This is also concordant with findings from a systematic review showing that individuals with PLEs are more than twice as likely to report mental health service use compared with those without PLEs (Bhavsar et al. 2018).

The clinical relevance of PLEs also originates from their association with suicide-related outcomes. Indeed, the majority of studies included in this systematic review that also addressed this point revealed that PLEs are directly related to the occurrence of suicidal ideation and behaviors as well as NSSI. Importantly, one study revealed that this association occurs only in people reporting high levels of insomnia (Misiak et al. 2024). Another cross-sectional study performed on university students also reported that the association of PLEs with suicidal ideation is significant in individuals with poor sleep quality but not those without sleep difficulties (Thompson et al. 2021). However, some cross-sectional studies have revealed that sleep disturbance mediates the association of PLEs with suicidal ideation (Farah et al. 2023; Luo et al. 2023). In turn, one longitudinal study found that PLEs mediate the association of sleep disturbance and short sleep duration with suicidal ideation (Bu et al. 2024). Because of a scarcity of evidence from longitudinal studies, conclusions about the temporal ordering of PLEs, sleep disturbance, and suicide risk are difficult to establish. Nevertheless, it is needed to note that both sleep disturbance, especially insomnia, and PLEs have been associated with suicide risk as demonstrated by previous meta-analyses (Liu et al. 2020; Yates et al. 2019). However, reported effect size estimates have been found small-to-moderate indicating potential doubts about the clinical relevance of the findings. Moreover, it has been noted that evidence of the association between sleep disturbance and suicide risk mostly originates from studies with longer follow-up periods (Liu et al. 2020). Also, little is known about how contextual factors contribute to observed effects.

It is further important to note that network analysis studies have provided grounds for understanding the association between a history of childhood trauma and PLEs. Importantly, a history of childhood trauma is known to be a risk factor for various mental disorders (McKay et al. 2021). However, some individuals appear to be resilient to its lasting consequences. Although some studies included in this systematic review revealed that childhood trauma history is directly related to the occurrence of PLEs, other studies also indicated important mediating mechanisms. For instance, Gaweda et al. (2021) revealed that sexual abuse leads to PLEs through other childhood adversities. Alternative pathways appeared to lead through cognitive biases and depressive symptoms. Similarly, general psychopathology, including depressive and anxiety symptoms, was found to mediate the association between childhood trauma and PLEs in the study by Qiao et al. (2024). These observations support the existence of previously observed effective pathways to psychosis (Myin-Germeys and van Os 2007). Indeed, it has been demonstrated that individuals at risk of psychosis show increased emotional reactivity to daily stressors. This phenomenon might also be attributed to exposure to childhood trauma (Lardinois et al. 2011).

### Methodological considerations

Several limitations across the included studies need to be considered. First, the majority of studies limited the assessment of PLEs to self-report measures. A lack of clinical validation might result in the recording of false positive findings. Nevertheless, it is interesting to note that self-reported PLEs representing false positive findings have also been shown to predict unfavorable mental health outcomes (Bak et al. 2003; van der Steen et al. 2019). Second, the majority of studies did not control for the effects of potential covariates, for example those related to sociodemographic characteristics. Third, the representativeness of cohorts assessed by specific studies might be limited. Fourth, there is considerable heterogeneity in reporting the results of a network analysis. Indeed, various measures of network stability and accuracy were not reported consistently. Similarly, only a minority of studies reported node predictability. Also, in some studies, specific measures of node centrality were used without sufficient rationale. For instance, it has been suggested that expected influence should be used for networks characterized by the presence of negative edges (Robinaugh et al. 2016). Fifth, a priori sample size calculations have not been performed by none of the included studies although some simulation-based approaches have been developed (Constantin et al. 2023). Finally, the majority of included studies were cross-sectional. Therefore, causality appears difficult to conclude.

### Limitations of a systematic review

There are some limitations at the level of a systematic review. First, no quantitative data synthesis was performed. This is because of the fact that a network analysis of PLEs was used to assess a variety of phenomena. Moreover, PLEs were not recorded using similar tools. Second, a formal quality assessment was not carried out using standardized tools for network analysis studies as such tools have not been developed so far. Third, a systematic review was limited to community samples. Therefore, the obtained results were not compared with those from clinical samples. However, it is important to note that PLEs are now perceived as non-specific psychopathological phenomena that occur in patients with various mental disorders. Fourth, it is needed to note some limitations of the approach to generalize findings with respect to the centrality of PLEs. Indeed, the centrality rank might be influenced by the number of nodes representing PLEs that differed largely between specific studies. For instance, the centrality rank of PLEs might be higher in networks that include a higher number of nodes representing PLEs compared with the number of nodes referring to other variables. Finally, it is needed to note that overlapping samples were analyzed by some studies. However, different hypotheses were tested by these studies.

### Implications for clinical practice

The recognition of PLEs might be important for clinical practice because of the fact that these phenomena are often associated with distress, impairment, and help-seeking behaviors. Moreover, PLEs are likely to appear in the context of various mental disorders. There is evidence that the presence of PLEs might be related to increased suicide risk. However, as shown in our systematic review, PLEs were not found to be ranked among the most central nodes in network analyses. Therefore, it is likely that interventions focused on other symptoms, likely those being the basis of psychiatric diagnosis or underlying psychological mechanisms might decrease the level of PLEs. In support of this claim, it should be noted that the intervention focused on improving resilience has been found to decrease the level of PLEs in college students with subclinical psychopathology (DeTore et al. 2023). In turn, cognitive-behavioral therapy might decrease the level of distress related to PLEs, but not the level of their occurrence (Soneson et al. 2020).

## Conclusions and future directions

Studies based on a network analysis have improved our understanding of PLEs, their nosological position, clinical relevance, and underlying mechanisms. However, certain aspects need to be pointed out to move the field forward. From a methodological point of view, it is needed to elaborate reporting protocols and tools to assess the quality of network analyses to improve the generalizability of findings. Also, clinical validation of reported symptoms is needed to increase the validity of observed associations.

The majority of included studies were cross-sectional and based on single measurements, and thus insights into potentially causal mechanisms are still limited. To address this point, it is not only needed to use a network analysis for longitudinal data but also implement this approach to real-life data obtained using the experience sampling method (ESM). The ESM refers to a variety of approaches that collect information about symptoms and behaviors in real-life environments, outside the laboratory setting (Myin-Germeys et al. 2018). The ESM studies show high ecological validity and enable to avoid the recall bias that is typical for single-timepoint, cross-sectional studies. The development of network analysis methods has offered opportunities to study the real-life dynamics of PLEs. Under this paradigm, three types of networks can be analyzed, that is the between-subjects network (undirected analysis illustrating the associations of variables across the whole sampling period), the contemporaneous network (undirected analysis that allows to assessment of concurrent associations within the same timeframe), and the temporal network (the analysis that allows to indicate direction of effects while controlling for autocorrelations) (Borsboom et al. 2021). Results of ESM studies may also result in the development of ecological momentary interventions for individuals with PLEs (Dao et al. 2021).

Another important direction for future studies is related to the need to use network analysis within a translational perspective. According to a network theory, the most central nodes might be considered optimal targets for interventions. Their activation (or deactivation) is most likely to increase (or decrease) the spread of information in the network. Previous studies have tested this hypothesis by investigating whether the most central nodes at baseline predict the onset and progression of psychopathology as well as treatment dropouts showing mixed findings (Boschloo et al. 2016; Groen et al. 2020; Lutz et al. 2018; Rodebaugh et al. 2018; Spiller et al. 2020). Importantly, one of these studies was included in the present systematic review and revealed that the most central nodes (i.e. depressive symptoms, negative affect, and loneliness) better predict follow-up PLEs (Qiao et al. 2024). Importantly, most of these studies investigated baseline cross-sectional networks that should be interpreted with caution because of a lack of insights into the temporal ordering of processes that give rise to the emergence of psychopathology (Bringmann et al. 2019). Network models can also be applied to analyze longitudinal data, including those recorded using ESM. In these models, two categories of node centrality might be analyzed, that is output centrality (the importance of nodes with respect to predicting other nodes over time) and input centrality (the importance of nodes with respect to the extent they are predicted by other nodes over time). Investigating output centrality may further inform the development of interventions for future testing using randomized clinical trials. Another translational direction for the field might be related to moving beyond group-level observations by developing person-specific networks (also known as idiographic networks) (Eaton et al. 2023). This approach might be integrated as a part of case conceptualization and the monitoring of therapeutic interventions. It might be particularly relevant for individuals with subclinical psychopathology that does not meet the clinical thresholds required to diagnose specific mental disorders. In these cases, person-specific networks covering risk, protective and maintenance factors, psychopathology, and various aspects of social functioning may provide insights into individual mechanisms underlying PLEs. However, existing evidence about person-centered networks is scarce and limited to feasibility studies (Fisher et al. 2017; Frumkin et al. 2021; Reeves and Fisher 2020; Riese et al. 2021; Rubel et al. 2018).

The development of network analysis approaches will also likely result in the inclusion of various types of data that fall beyond the assessment of psychopathological symptoms, hypothesized psychological mechanisms, and behaviors. To provide insights into the biopsychosocial contexts of PLEs, it will be needed to combine neuroimaging findings, physiological responses, and other biomarkers. However, although a network analysis does not impose a specific model of causality, the decision to include specific variables should be based on the theory that justifies their selection. Moreover, it is needed to note that increasing the number of variables but not including a higher number of participants and timepoints (in the case of longitudinal data) may decrease the network accuracy (Bringmann 2024).

In sum, a network analysis seems to be an important approach that allows us to understand the complexity of mechanisms underlying the emergence of PLEs, and develop and test novel interventions. However, progress in the field requires further application of network models to longitudinal data representing psychopathological manifestation, social functioning, risk, and protective factors as well as potential mechanisms. Moreover, progress is needed at the level of testing the clinical usefulness of person-specific networks to better inform case conceptualization in the case of individuals with PLEs. Lessons provided by a critical appraisal of existing evidence can improve designing future studies of PLEs based on network analysis.

## Supporting information

Misiak et al. supplementary material Misiak et al. supplementary material

## Supplementary material

To view supplementary material for this article, please visit http://doi.org/10.1017/S0033291725000261.

## References

[r1] Adjorlolo, S., Anum, A., & Adjorlolo, P. (2021) Latent structure of psychotic-like experiences in adolescents: Evidence from a multi-method taxometric study of a school-based sample in Ghana. Psychiatry Researchearch 302, 113991. 10.1016/j.psychres.2021.113991. 34029985

[r2] Astill Wright, L., McElroy, E., Barawi, K., Roberts, N. P., Simon, N., Zammit, S., & Bisson, J. I. (2023) Associations among psychosis, mood, anxiety, and posttraumatic stress symptoms: A network analysis. Journal of Traumatic Stress 36(2), 385–396. 10.1002/jts.22916. 36862599

[r3] Bak, M., Delespaul, P., Hanssen, M., de Graaf, R., Vollebergh, W., & van Os, J. (2003) How false are “false” positive psychotic symptoms? Schizophrenia Research 62(1–2), 187–189. 10.1016/s0920-9964(02)00336-5. 12765760

[r4] Bebbington, P., & Nayani, T. (1995) The Psychosis Screening Questionnaire. Int J Methods Psychiatry Researchearch 5(1), 11–19.

[r5] Bell, V., Halligan, P. W., & Ellis, H. D. (2006) The Cardiff Anomalous Perceptions Scale (CAPS): a new validated measure of anomalous perceptual experience. Schizophrenia Bulletin 32(2), 366–377. 10.1093/schbul/sbj014. 16237200 PMC2632213

[r6] Betz, L. T., Penzel, N., Rosen, M., Bhui, K., Upthegrove, R., & Kambeitz, J. (2023) Disentangling heterogeneity of psychosis expression in the general population: sex-specific moderation effects of environmental risk factors on symptom networks. Psychological Medicine 53(5), 1860–1869. 10.1017/S0033291721003470. 37310332

[r7] Bhavsar, V., McGuire, P., MacCabe, J., Oliver, D., & Fusar-Poli, P. (2018) A systematic review and meta-analysis of mental health service use in people who report psychotic experiences. Early Intervention in Psychiatry 12(3), 275–285. 10.1111/eip.12464. 28805304 PMC6001621

[r8] Borsboom, D., Deserno, M. K., Rhemtulla, M., Epskamp, S, Fried, E. I., McNally, R. J., … Waldorp, L. J. (2021) Network analysis of multivariate data in psychological science. Nature Reviews Methods Primers 1(1), 58. 10.1038/s43586-021-00055-w.

[r9] Boschloo, L., van Borkulo, C. D., Borsboom, D., & Schoevers, R. A. (2016) A Prospective Study on How Symptoms in a Network Predict the Onset of Depression. Psychotherapy and Psychosomatics 85(3), 183–184. 10.1159/000442001. 27043457

[r10] Bringmann, L. F., Elmer, T., Epskamp, S., Krause, R. W., Schoch, D., Wichers, M., … Snippe, E. (2019) What do centrality measures measure in psychological networks? Journal of Abnormal Psychology 128(8), 892–903. 10.1037/abn0000446. 31318245

[r11] Bringmann, L. F. (2024) The future of dynamic networks in research and clinical practice. World Psychiatry 23(2), 288–289. 10.1002/wps.21209. 38727056 PMC11083907

[r12] Bu, L., Wang, D., Fan, Y., Ye, H., Liu, W., & Fan, F. (2024) Sleep disturbance and suicidal ideation mediated by psychotic-like experiences in adolescents: a two-wave longitudinal study. Sleep 47(3):zsae013. 10.1093/sleep/zsae013. 38230742

[r13] Cernis, E., Evans, R., Ehlers, A., & Freeman, D. (2021) Dissociation in relation to other mental health conditions: An exploration using network analysis. Journal of Psychiatric Research 136, 460–467. 10.1016/j.jpsychires.2020.08.023. 33092867 PMC8039185

[r14] Cheng, P., Liu, Z., Sun, M., Zhang, W., Guo, R., Hu, A., & Long, Y. (2024) The relations of psychotic-like experiences (PLEs) and depressive symptoms and the bias of depressive symptoms during the clustering among Chinese adolescents: Findings from the network perspective. Journal of Affective Disorders 350, 867–876. 10.1016/j.jad.2024.01.180. 38272370

[r15] Constantin, M. A., Schuurman, N. K., & Vermunt, J. K. (2023) A general Monte Carlo method for sample size analysis in the context of network models. Psychological Methods 10.1037/met0000555. 37428726

[r16] Costello, E. J., Edelbrock, C. S., & Costello, A. J. (1985) Validity of the NIMH Diagnostic Interview Schedule for Children: a comparison between psychiatric and pediatric referrals. Journal of Abnormal Child Psychology 13(4), 579–595. 10.1007/BF00923143. 4078188

[r17] Cuesta, M. J., Ugarte, M. D., Goicoa, T., Eraso, S., & Peralta, V. (2007) A taxometric analysis of schizophrenia symptoms. Psychiatry Researchearch 150(3), 245–253. 10.1016/j.psychres.2006.01.019. 17316823

[r18] Dao, K. P., De Cocker, K., Tong, H. L., Kocaballi, A. B., Chow, C., & Laranjo, L. (2021) Smartphone-Delivered Ecological Momentary Interventions Based on Ecological Momentary Assessments to Promote Health Behaviors: Systematic Review and Adapted Checklist for Reporting Ecological Momentary Assessment and Intervention Studies. JMIR Mhealth and Uhealth 9(11), e22890. 10.2196/22890. 34806995 PMC8663593

[r19] Deng, W., Everaert, J., Bronstein, M. V., Joormann, J., & Cannon, T. (2023) Social Interpretation Inflexibility and Functioning: Associations with Symptoms and Stress. Journal of Social and Clinical Psychology 42(1), 29–49. 10.1521/jscp.2023.42.1.29.

[r20] DeTore, N. R., Luther, L., Deng, W., Zimmerman, J., Leathem, L., Burke, A. S., … Holt, D. J. (2023) Efficacy of a transdiagnostic, prevention-focused program for at-risk young adults: a waitlist-controlled trial. Psychological Medicine 53(8), 3490–3499. 10.1017/S0033291722000046. 35227342 PMC9433469

[r23] Eaton, N. R., Bringmann, L. F., Elmer, T., Fried, E. I., Forbes, M. K., Greene, A. L., … Waszczuk, M. A. (2023) A review of approaches and models in psychopathology conceptualization research. Nature Reviews Psychology 2, 622–636.

[r24] Elahi, A., Perez Algorta, G., Varese, F., McIntyre, J. C., & Bentall, R. P. (2017) Do paranoid delusions exist on a continuum with subclinical paranoia? A multi-method taxometric study. Schizophrenia Research 190, 77–81. 10.1016/j.schres.2017.03.022. 28318838

[r25] Epskamp, S., Borsboom, D., & Fried, E. I. (2018a) Estimating psychological networks and their accuracy: A tutorial paper. Behavior Research Methods 50(1), 195–212. 10.3758/s13428-017-0862-1. 28342071 PMC5809547

[r26] Epskamp, S., Waldorp, L. J., Mottus, R., & Borsboom, D. (2018b) The Gaussian Graphical Model in Cross-Sectional and Time-Series Data. Multivariate Behavioral Research 53(4), 453–480. 10.1080/00273171.2018.1454823. 29658809

[r27] Farah, N., Obeid, S., Malaeb, D., Haddad, C., Fekih-Romdhane, F., & Hallit, S. (2023) Mediation effect of insomnia symptoms between positive psychotic like experiences and suicidal ideation among Lebanese young adults. BMC Psychiatry 23(1):272. 10.1186/s12888-023-04778-w. 37081441 PMC10116113

[r28] Feczko, E., Miranda-Dominguez, O., Marr, M., Graham, A. M., Nigg, J. T., & Fair, D. A. (2019) The Heterogeneity Problem: Approaches to Identify Psychiatric Subtypes. Trends in Cognitive Sciences 23(7), 584–601. 10.1016/j.tics.2019.03.009. 31153774 PMC6821457

[r29] Fisher, A. J., Reeves, J. W., Lawyer, G., Medaglia, J. D., & Rubel, J. A. (2017) Exploring the idiographic dynamics of mood and anxiety via network analysis. Journal of Abnormal Psychology 126, 1044–1056. 10.1037/abn0000311. 29154565

[r30] Fonseca-Pedrero, E., Muniz, J., Gacia-Portilla, M. P., & Bobes, J. (2021) Network structure of psychotic-like experiences in adolescents: Links with risk and protective factors. Early Intervention in Psychiatry 15(3), 595–605. 10.1111/eip.12989. 32419341

[r31] Freeman, D., Loe, B. S., Kingdon, D., Startup, H., Molodynski, A., Rosebrock, L., … Bird, J. C. (2021) The revised Green et al., Paranoid Thoughts Scale (R-GPTS): psychometric properties, severity ranges, and clinical cut-offs. Psychological Medicine 51(2), 244–253. 10.1017/S0033291719003155. 31744588 PMC7893506

[r32] Frumkin, M. R., Piccirillo, M. L., Beck, E. D., Grossman, J. T. & Rodebaugh, T. L. (2021) Feasibility and utility of idiographic models in the clinic: A pilot study. Psychotherapy Research 31, 520–534. 10.1080/10503307.2020.1805133. 32838671 PMC7902742

[r33] Fung, H. W., Wong, M. Y. C., Moskowitz, A., Chien, W. T., Hung, S. L., & Lam, S. K. K. (2024) Association Between Psychotic and Dissociative Symptoms: Further Investigation Using Network Analysis. Journal of Trauma and Dissociation 25(2), 279–296. 10.1080/15299732.2023.2293776. 38124492

[r34] Fusar-Poli, P., Solmi, M., Brondino, N., Davies, C., Chae, C., Politi, P., … McGuire, P. (2019) Transdiagnostic psychiatry: a systematic review. World Psychiatry 18(2):192–207. 10.1002/wps.20631. 31059629 PMC6502428

[r35] Gaweda, L., Kowalski, J., Aleksandrowicz, A., Bagrowska, P., Dabkowska, M., & Pionke-Ubych, R. (2024) A systematic review of performance-based assessment studies on cognitive biases in schizophrenia spectrum psychoses and clinical high-risk states: A summary of 40 years of research. Clinical Psychology Revies 108, 102391. 10.1016/j.cpr.2024.102391. 38301343

[r36] Gaweda, L., Pionke, R., Hartmann, J., Nelson, B., Cechnicki, A., & Frydecka, D. (2021) Toward a Complex Network of Risks for Psychosis: Combining Trauma, Cognitive Biases, Depression, and Psychotic-like Experiences on a Large Sample of Young Adults. Schizophrenia Bulletin 47(2), 395–404. 10.1093/schbul/sbaa125. 33728467 PMC7965064

[r37] Giocondo, J. G., Salum, G. A., Gadelha, A., Argolo, F. C., Simioni, A. R., Mari, J. J., … Pan, P. M. (2021) Psychotic-like Experiences and Common Mental Disorders in Childhood and Adolescence: Bidirectional and Transdiagnostic Associations in a Longitudinal Community-based Study. Schizophrenia Bulletin Open 2(1), sgab028. 10.1093/schizbullopen/sgab028.

[r38] Grant, B. F., Dawson, D. A., Stinson, F. S., Chou, P. S., Kay, W., & Pickering, R. (2003) The Alcohol Use Disorder and Associated Disabilities Interview Schedule-IV (AUDADIS-IV): reliability of alcohol consumption, tobacco use, family history of depression and psychiatric diagnostic modules in a general population sample. Drug and Alcohol Dependence 71(1), 7–16. 10.1016/s0376-8716(03)00070-x. 12821201

[r39] Groen, R. N., Ryan, O., Wigman, J. T. W., Riese, H., Penninx, B. W. J. H., Giltay, E. J., … Hartman, C. A. (2020) Comorbidity between depression and anxiety: assessing the role of bridge mental states in dynamic psychological networks. BMC Medicine 18(1), 308. 10.1186/s12916-020-01738-z. 32988400 PMC7523307

[r40] Hajduk, M., Strakova, A., Januska, J., Ivancik, V., Dancik, D., Cavojska, N., … Green, M. F. (2023) Connections between and within extended psychosis and autistic phenotypes and social relationships in the general population. Journal of Psychiatric Research 157, 36–42. 10.1016/j.jpsychires.2022.11.022. 36436426

[r41] Haslbeck, J. M. B., & Fried, E. I. (2017) How predictable are symptoms in psychopathological networks? A reanalysis of 18 published datasets. Psychological Medicine 47(16), 2767–2776. 10.1017/S0033291717001258. 28625186

[r42] Haslbeck, J. M. B., Ryan, O., Robinaugh, D. J., Waldorp, L. J., & Borsboom, D. (2022) Modeling psychopathology: From data models to formal theories. Psychological Methods 27(6), 930–957. 10.1037/met0000303. 34735175 PMC10259162

[r43] Haslbeck, J. M. B., & Waldorp, L. J. (2020) MGM: Estimating time-varying mixed graphical models in high-dimensional data. Journal of Statistical Software 93(8), 1–46.

[r44] Healy, C., Brannigan, R., Dooley, N., Coughlan, H., Clarke, M., Kelleher, I., & Cannon, M. (2019) Childhood and adolescent psychotic experiences and risk of mental disorder: a systematic review and meta-analysis. Psychological Medicine 49(10), 1589–1599. 10.1017/S0033291719000485. 31088578

[r45] Hinterbuchinger, B., & Mossaheb, N. (2021) Psychotic-Like Experiences: A Challenge in Definition and Assessment. Frontiers in Psychiatry 12, 582392. 10.3389/fpsyt.2021.582392. 33854445 PMC8039445

[r46] Huang, Y. H., Hu, H. X., Wang, L. L., Zhang, Y. J., Wang, X., Wang, Y., … Chan, R. C. K. (2023) Relationships between childhood trauma and dimensional schizotypy: A network analysis and replication. Asian Journal of Psychiatry 85, 103598. 10.1016/j.ajp.2023.103598. 37119684

[r47] Ising, H. K., Veling, W., Loewy, R. L., Rietveld, M. W., Rietdijk, J., Dragt, S., … van der Gaag, M. (2012) The validity of the 16-item version of the Prodromal Questionnaire (PQ-16) to screen for ultra high risk of developing psychosis in the general help-seeking population. Schizophrenia Bulletin 38(6), 1288–1296. 10.1093/schbul/sbs068. 22516147 PMC3713086

[r48] Jablensky, A. (2016) Psychiatric classifications: validity and utility. World Psychiatry 15(1), 26–31. 10.1002/wps.20284. 26833601 PMC4780305

[r49] Jones, P. J., Ma, R., & McNally, R. J. (2021) Bridge Centrality: A Network Approach to Understanding Comorbidity. Multivariate Behavior Research 56(2), 353–367. 10.1080/00273171.2019.1614898. 31179765

[r50] Jones, P. J., Mair, P., Simon, T., & Zeileis, A. (2020) Network Trees: A Method for Recursively Partitioning Covariance Structures. Psychometrika 85(4), 926–945. 10.1007/s11336-020-09731-4. 33146786

[r51] Kelleher, I., & Cannon, M. (2011) Psychotic-like experiences in the general population: characterizing a high-risk group for psychosis. Psychological Medicine 41(1):1–6. 10.1017/S0033291710001005. 20624328

[r52] Kendell, R., & Jablensky, A. (2003) Distinguishing between the validity and utility of psychiatric diagnoses. American Journal of Psychiatry 160(1), 4–12. 10.1176/appi.ajp.160.1.4. 12505793

[r53] Kobayashi, H., Nemoto, T., Koshikawa, H., Osono, Y., Yamazawa, R., Murakami, M., … Mizuno, M. (2008) A self-reported instrument for prodromal symptoms of psychosis: testing the clinical validity of the PRIME Screen-Revised (PS-R) in a Japanese population. Schizophrenia Research 106(2–3), 356–362. 10.1016/j.schres.2008.08.018. 18809299

[r54] Kwapil, T. R., Gross, G. M., Silvia, P. J., Raulin, M. L., & Barrantes-Vidal, N. (2018) Development and psychometric properties of the Multidimensional Schizotypy Scale: A new measure for assessing positive, negative, and disorganized schizotypy. Schizophrenia Research 193, 209–217. 10.1016/j.schres.2017.07.001. 28735642

[r55] Lardinois, M., Lataster, T., Mengelers, R., Van Os, J., & Myin-Germeys, I. (2011) Childhood trauma and increased stress sensitivity in psychosis. Acta Psychiatrica Scandinavica 123(1), 28–35. 10.1111/j.1600-0447.2010.01594.x. 20712824

[r56] Lindgren, M., Numminen, L., Holm, M., Therman, S., & Tuulio-Henriksson, A. (2022) Psychotic-like experiences of young adults in the general population predict mental disorders. Psychiatry Research 312, 114543. 10.1016/j.psychres.2022.114543. 35417824

[r57] Liu, R. T., Steele, S. J., Hamilton, J. L., Do, Q. B. P., Furbish, K., Burke, T. A., … Gerlus, N. (2020) Sleep and suicide: A systematic review and meta-analysis of longitudinal studies. Clinical Psychology Reviews 81, 101895. 10.1016/j.cpr.2020.101895. PMC7731893 32801085

[r58] Livet, A., Navarri, X., Potvin, S., & Conrod, P. (2020) Cognitive biases in individuals with psychotic-like experiences: A systematic review and a meta-analysis. Schizophrenia Research 222:10–22. 10.1016/j.schres.2020.06.016. 32595098

[r59] Loewy, R. L., Pearson, R., Vinogradov, S., Bearden, C. E., & Cannon, T. D. (2011) Psychosis risk screening with the Prodromal Questionnaire--brief version (PQ-B). Schizophrenia Research 129(1), 42–46. 10.1016/j.schres.2011.03.029. 21511440 PMC3113633

[r60] Luo, X., Yu, T., Yang, Z., & Wang, D. (2023) Psychotic-Like Experiences and Suicidal Ideation Among Adolescents: The Chain Mediating Role of Insomnia Symptoms and Resilience. Psychology Research and Behavior Management 16, 3519–3530. 10.2147/PRBM.S426363. 37675191 PMC10478937

[r61] Lutz, W., Schwartz, B., Hofmann, S. G., Fisher, A. J., Husen, K., & Rubel, J. A. (2018) Using network analysis for the prediction of treatment dropout in patients with mood and anxiety disorders: A methodological proof-of-concept study. Scientific Reports 8(1), 7819. 10.1038/s41598-018-25953-0. 29777110 PMC5959887

[r62] McGrath, J. J., Saha, S., Al-Hamzawi, A., Alonso, J., Bromet, E. J., Bruffaerts, R., … Kessler, R. C. (2015) Psychotic Experiences in the General Population: A Cross-National Analysis Based on 31,261 Respondents From 18 Countries. JAMA Psychiatry 72(7), 697–705. 10.1001/jamapsychiatry.2015.0575. 26018466 PMC5120396

[r63] McKay, M. T., Cannon, M., Chambers, D., Conroy, R. M., Coughlan, H., Dodd, P., Healy, C., … Clarke, M. C. (2021) Childhood trauma and adult mental disorder: A systematic review and meta-analysis of longitudinal cohort studies. Acta Psychiatrica Scandinavica 143(3), 189–205. 10.1111/acps.13268. 33315268

[r64] Miller, T. J., McGlashan, T. H., Rosen, J. L., Cadenhead, K., Cannon, T., Ventura, J., … Woods, S. W. (2003) Prodromal assessment with the structured interview for prodromal syndromes and the scale of prodromal symptoms: predictive validity, interrater reliability, and training to reliability. Schizophrenia Bulletin 29(4), 703–715. 10.1093/oxfordjournals.schbul.a007040. 14989408

[r65] Misiak, B., & Frydecka, D. (2024) Psychotic-like experiences predict the perceived intent to seek treatment: A network perspective. Schizophrenia Research 266, 100–106. 10.1016/j.schres.2024.02.033. 38387252

[r66] Misiak, B., Gaweda, L., Moustafa, A. A., & Samochowiec, J. (2024) Insomnia moderates the association between psychotic-like experiences and suicidal ideation in a non-clinical population: a network analysis. European Archives of Psychiatry and Clinical Neuroscience 274(2), 255–263. 10.1007/s00406-023-01653-3. 37516979 PMC10914899

[r67] Misiak, B., Samochowiec, J., Kowalski, K., Gaebel, W., Bassetti, C. L. A., Chan, A., … Falkai, P. (2023a) The future of diagnosis in clinical neurosciences: Comparing multiple sclerosis and schizophrenia. European Psychiatry 66(1), e58. 10.1192/j.eurpsy.2023.2432. 37476977 PMC10486256

[r68] Misiak, B., Kowalski, K., Jaworski, A., Swirkosz, G., Szyszka, M., & Piotrowski P (2023b) Understanding pathways from narcissistic grandiosity to psychotic-like experiences: Insights from the network analysis. Journal of Psychiatric Research 166, 122–129. 10.1016/j.jpsychires.2023.09.019. 37757705

[r69] Misiak, B., Szewczuk-Boguslawska, M., Samochowiec, J., Moustafa, A. A., & Gawęda Ł (2023c) Unraveling the complexity of associations between a history of childhood trauma, psychotic-like experiences, depression and non-suicidal self-injury: A network analysis. Journal of Affective Disorders. 10.1016/j.jad.2023.05.044. 37230261

[r70] Murphy, J., McBride. O., Fried, E., & Shevlin, M. (2018) Distress, Impairment and the Extended Psychosis Phenotype: A Network Analysis of Psychotic Experiences in an US General Population Sample. Schizophrenia Bulletin 44(4), 768–777. 10.1093/schbul/sbx134. 29036519 PMC6007708

[r71] Myin-Germeys, I., Kasanova, Z., Vaessen, T., Vachon, H., Kirtley, O., Viechtbauer, W., & Reininghaus, U. (2018) Experience sampling methodology in mental health research: new insights and technical developments. World Psychiatry 17(2), 123–132. 10.1002/wps.20513. 29856567 PMC5980621

[r72] Myin-Germeys, I., & van Os, J. (2007) Stress-reactivity in psychosis: evidence for an affective pathway to psychosis. Clinical Psychology Reviews 27(4), 409–424. 10.1016/j.cpr.2006.09.005. 17222489

[r73] Nunez, D., Fresno, A., van Borkulo, C. D., Courtet, P., Arias, V., Garrido, V., & Wigman, J. T. W. (2018) Examining relationships between psychotic experiences and suicidal ideation in adolescents using a network approach. Schizophrenia Research 201, 54–61. 10.1016/j.schres.2018.05.020. 29804930

[r74] Nunez, D., Monjes, P., Campos, S., & Wigman, J. T. W. (2020) Evidence for Specific Associations Between Depressive Symptoms, Psychotic Experiences, and Suicidal Ideation in Chilean Adolescents From the General Population. Frontiers in Psychiatry 11, 552343. 10.3389/fpsyt.2020.552343. 33584356 PMC7876080

[r75] Page, M.J., McKenzie, J.E., Bossuyt, P. M., Boutron, I., Hoffmann, T. C., Mulrow, C. D., … Moher, D. (2021) The PRISMA 2020 statement: an updated guideline for reporting systematic reviews. British Medical Journal 372, n71. 10.1136/bmj.n71. 33782057 PMC8005924

[r76] Qiao, Z., Lafit, G., Lecei, A., Achterhof, R., Kirtley, O. J., Hiekkaranta, A. P., … van Winkel, R. (2024) Childhood Adversity and Emerging Psychotic Experiences: A Network Perspective. Schizophrenia Bulletin 50(1), 47–58. 10.1093/schbul/sbad079. 37318106 PMC10754171

[r77] Rejek, M., & Misiak, B. (2023) Dimensions of psychopathology associated with psychotic-like experiences: Findings from the network analysis in a nonclinical sample. European Psychiatry 66(1), e56. 10.1192/j.eurpsy.2023.2429. 37439195 PMC10486255

[r78] Rejek, M., & Misiak, B. (2024) Modelling the effects of the exposome score within the extended psychosis phenotype. Journal of Psychiatric Research 169, 22–30. 10.1016/j.jpsychires.2023.11.022. 37995498

[r79] Reeves, J. W., & Fisher, A. J. (2020) An Examination of Idiographic Networks of Posttraumatic Stress Disorder Symptoms. Journal of Traumatic Stress 33(1), 84–95. 10.1002/jts.22491. 32103567

[r80] Riese, H., von Klipstein, L., Schoevers, R. A., van der Veen, D. C., & Servaas, M. N. (2021) Personalized ESM monitoring and feedback to support psychological treatment for depression: a pragmatic randomized controlled trial (Therap-i). BMC Psychiatry 21(1), 143. 10.1186/s12888-021-03123-3. 33691647 PMC7945664

[r81] Robinaugh, D. J., Hoekstra, R. H. A., Toner, E.R., & Borsboom, D. (2020) The network approach to psychopathology: a review of the literature 2008-2018 and an agenda for future research. Psychological Medicine 50(3), 353–366. 10.1017/S0033291719003404. 31875792 PMC7334828

[r82] Robinaugh, D. J., Millner, A. J, & McNally, R. J. (2016) Identifying highly influential nodes in the complicated grief network. Journal of Abnormal Psychology 125(6), 747–757. 10.1037/abn0000181. 27505622 PMC5060093

[r83] Rodebaugh, T. L., Tonge, N. A., Piccirillo, M. L., Fried, E., Horenstein, A., Morrison, A. S., … Heimberg, R. G. (2018) Does centrality in a cross-sectional network suggest intervention targets for social anxiety disorder? Journal of Consulting and Clinical Psychology 86(10), 831–844. 10.1037/ccp0000336. 30265042 PMC6166439

[r84] Ronald, A., Sieradzka, D., Cardno, A. G., Haworth, C. M., McGuire, P., & Freeman, D. (2014) Characterization of psychotic experiences in adolescence using the specific psychotic experiences questionnaire: findings from a study of 5000 16-year-old twins. Schizophrenia Bulletin 40(4), 868–877. 10.1093/schbul/sbt106. 24062593 PMC4059437

[r85] Rubel, J.A., Fisher, A. J., Husen, K., & Lutz, W. (2018) Translating Person-Specific Network Models into Personalized Treatments: Development and Demonstration of the Dynamic Assessment Treatment Algorithm for Individual Networks (DATA-IN). Psychotherapy and Psychosomatics 87(4), 249–251. 10.1159/000487769. 29680835

[r86] Scott, J., Crouse, J. J., Ho, N., Carpenter, J., Martin, N., Medland, S., … Hickie, I. (2021) Can network analysis of self-reported psychopathology shed light on the core phenomenology of bipolar disorders in adolescents and young adults? Bipolar Disorders 23(6), 584–594. 10.1111/bdi.13067. 33638252 PMC8387492

[r87] Soneson, E., Russo, D., Stochl, J., Heslin, M., Galante, J., Knight, C., … Perez, J. (2020) Psychological interventions for people with psychotic experiences: A systematic review and meta-analysis of controlled and uncontrolled effectiveness and economic studies. Australian and New Zeland Journal of Psychiatry 54(7), 673–695. 10.1177/0004867420913118. PMC7324911 32462893

[r88] Spiller, T. R., Levi, O., Neria, Y., Suarez-Jimenez, B., Bar-Haim, Y., & Lazarov, A. (2020) On the validity of the centrality hypothesis in cross-sectional between-subject networks of psychopathology. BMC Medicine 18(1), 297. 10.1186/s12916-020-01740-5. 33040734 PMC7549218

[r89] Staines, L., Healy, C., Murphy, F., Byrne, J., Murphy, J., Kelleher, I., … Cannon, M. (2023) Incidence and Persistence of Psychotic Experiences in the General Population: Systematic Review and Meta-Analysis. Schizophrenia Bulletin. 10.1093/schbul/sbad056. PMC10318880 37402250

[r90] Staines, L., Healy, C., Coughlan, H., Clarke, M., Kelleher, I., Cotter, D., & Cannon M (2022) Psychotic experiences in the general population, a review; definition, risk factors, outcomes and interventions. Psychological Medicine 52(15), 1–12. 10.1017/S0033291722002550. PMC9772919 36004805

[r91] Steenkamp, L. R., de Neve-Enthoven, N. G. M., João, A. M., Bouter, D. C., Hillegers, M. H. J., Hoogendijk, W. J. G., … Bolhuis, K. (2023) Psychotic experiences, suicidality and non-suicidal self-injury in adolescents: Independent findings from two cohorts. Schizophrenia Research 257, 50–57. 10.1016/j.schres.2023.05.006. 37285715

[r92] Stefanis, N. C., Hanssen, M., Smirnis, N. K., Avramopoulos, D. A., Evdokimidis, I. K., Stefanis, C. N., … Van Os, J. (2002) Evidence that three dimensions of psychosis have a distribution in the general population. Psychological Medicine 32(2), 347–358. 10.1017/s0033291701005141. 11866327

[r93] Suen, Y. N., Chau, A. P. Y., Wong, S. M. Y., Hui, C. L. M., Chan, S. K. W., Lee, E. H. M., … Chen, E. Y. H. (2024) Comorbidity of autism spectrum and attention deficit/hyperactivity disorder symptoms and their associations with 1-year mental health outcomes in adolescents and young adults. Psychiatry Research 331, 115657. 10.1016/j.psychres.2023.115657. 38056129

[r94] Sullivan, S. A., Kounali, D., Cannon, M., David, A. S., Fletcher, P. C., Holmans, P., … Zammit, S. (2020) A Population-Based Cohort Study Examining the Incidence and Impact of Psychotic Experiences From Childhood to Adulthood, and Prediction of Psychotic Disorder. American Journal of Psychiatry 177(4), 308–317. 10.1176/appi.ajp.2019.19060654. 31906710

[r95] Sun, X., & Zhong, J. (2023) The dimensionality of perceptual anomalies and their relationships with bullying victimization among Chinese adolescents: From a network perspective. Schizophrenia Research 262, 42–50. 10.1016/j.schres.2023.10.031. 37922843

[r96] Taylor, M. J., Freeman, D., & Ronald, A. (2016) Dimensional psychotic experiences in adolescence: Evidence from a taxometric study of a community-based sample. Psychiatry Research 241, 35–42. 10.1016/j.psychres.2016.04.021. 27155285 PMC4922386

[r97] Thompson, E. C., Jay, S. Y., Andorko, N. D., Millman, Z. B., Rouhakhtar, P. R., Sagun, K., … Schiffman. J. (2021) Sleep quality moderates the association between psychotic-like experiences and suicidal ideation among help-seeking university students. Psychiatry Research 296, 113668. 10.1016/j.psychres.2020.113668. 33401091 PMC8482876

[r98] Valle, R. (2022) Validity, reliability and clinical utility of mental disorders: The case of ICD-11 schizophrenia. Revista Colombiana de Psiquiatria (English Edition) 51(1), 61–70. 10.1016/j.rcpeng.2020.09.003. 35210207

[r99] van Borkulo, C., Boschloo, L., Borsboom, D., Penninx, B. W., Waldorp, L. J., & Schoevers, R. A. (2015) Association of Symptom Network Structure With the Course of Depression. JAMA Psychiatry 72(12), 1219–1226. 10.1001/jamapsychiatry.2015.2079. 26561400

[r100] van Borkulo, C. D., van Bork, R., Boschloo, L., Kossakowski, J. J., Tio, P., Schoevers, R. A., … Waldorp, L. J. (2022) Comparing network structures on three aspects: A permutation test. Psychological Methods. 10.1037/met0000476. 35404628

[r101] van der Steen, Y., Myin-Germeys, I., van Nierop, M., Ten Have, M., de Graaf, R., van Dorsselaer, S., … van Winkel, R. (2019) ‘False-positive’ self-reported psychotic experiences in the general population: an investigation of outcome, predictive factors and clinical relevance. Epidemiology and Psychiatric Sciences 28(5), 532–543. 10.1017/S2045796018000197. 29656729 PMC6998918

[r102] van Os, J., Linscott, R. J., Myin-Germeys, I., Delespaul, P., & Krabbendam, L. (2009) A systematic review and meta-analysis of the psychosis continuum: evidence for a psychosis proneness-persistence-impairment model of psychotic disorder. Psychological Medicine 39(2), 179–195. 10.1017/S0033291708003814. 18606047

[r109] Wüsten, C., Schlier, B., Jaya, E. S., Genetic Risk and Outcome of Psychosis (GROUP) Investigators, Fonseca-Pedrero, E., Peters, E., … Lincoln TM. (2018) Psychotic Experiences and Related Distress: A Cross-national Comparison and Network Analysis Based on 7141 Participants From 13 Countries. Schizophrenia Bulletin 44(6), 1185–1194. 10.1093/schbul/sby087. 29982814 PMC6192474

[r103] Xavier, R. M., Calkins, M. E., Bassett, D. S., Moore, T. M., George, W. T., Taylor, J. H., & Gur, R. E. (2022) Characterizing Youth-Caregiver Concordance and Discrepancies in Psychopathology Symptoms in a US Community Sample. Issues in Mental Health Nursing 43(11), 1004–1013. 10.1080/01612840.2022.2099494. 35839118 PMC9709771

[r104] Yang, X. H., Zhang, J. W., Li, Y., Zhou, L., & Sun, M. (2023) Psychotic-like experiences as a co-occurring psychopathological indicator of multi-dimensional affective symptoms: Findings from a cross-sectional survey among college students. Journal of Affective Disorders 323, 33–39. 10.1016/j.jad.2022.11.053. 36435396

[r105] Yates, K., Lang, U., Cederlof, M., Boland, F., Taylor, P., Cannon, M., … Kelleher, I. (2019) Association of Psychotic Experiences With Subsequent Risk of Suicidal Ideation, Suicide Attempts, and Suicide Deaths: A Systematic Review and Meta-analysis of Longitudinal Population Studies. JAMA Psychiatry 76(2), 180–189. 10.1001/jamapsychiatry.2018.3514. 30484818 PMC6439738

[r106] Yung, A. R., Nelson, B., Baker, K., Buckby, J. A., Baksheev, G., & Cosgrave, E. M. (2009) Psychotic-like experiences in a community sample of adolescents: implications for the continuum model of psychosis and prediction of schizophrenia. Australian and New Zeland Journal of Psychiatry 43(2), 118–128. 10.1080/00048670802607188. 19153919

[r107] Zhou, H. Y., Luo, Y. H., Shi, L. J., & Gong, J. (2023) Exploring psychological and psychosocial correlates of non-suicidal self-injury and suicide in college students using network analysis. Journal of Affective Disorders 336, 120–125. 10.1016/j.jad.2023.05.089. 37257782

[r108] Zhou, R., Foo, J. C., Nishida, A., Ogawa, S., Togo, F., & Sasaki, T. (2024) Longitudinal relationships of psychotic-like experiences with suicidal ideation and self-harm in adolescents. European Child and Adolescent Psychiatry. 33(6), 1977–1985. 10.1007/s00787-023-02299-1. 37740799 PMC11211151

