# Supplementary material for: A systematic review of studies using network analysis to assess dynamics of psychotic-like experiences in community samples
Source: Psychol Med. 2025 Feb 19;55:e54. doi: 10.1017/S0033291725000261 (PMC12080667; doi:10.1017/S0033291725000261)
Supplement: Misiak et al. supplementary material [file S0033291725000261sup001.docx]

**Supplementary Appendix**

**Table 1.** Centrality ranks of nodes representing PLEs.

|  | N | Strength | Betweenness | Closeness | Expected  Influence | Bridge Expected Influence |
| --- | --- | --- | --- | --- | --- | --- |
| Cernis et al. (2021) | 11 | 6 (54.5) | 6 (54.5) | 7 (63.6) | - | - |
| Cheng et al. (2024) | 42 | - | - | - | 2 (4.8) | 10 (23.8) |
| Deng et al. (2023) | 7 | - | - | - | - | - |
| Fonseca-Pedrero et al. (2021) | 11 | - | - | - | 1 (9.1) | - |
| Fung et al. (2024) | 18 | 6 (33.3) | 4 (22.2) | 2 (11.1) | - | - |
| Gaweda et al. (2021) | 34 | 7 (20.6) | - | - | - | - |
| Hajduk et al. (2023) | 21 | 3 (14.3) | - | - | 3 (14.3) | - |
| Huang et al. (2023) | 14 | 4 (28.6) | 5 (35.7) | 2 (14.3) | 3 (21.4) | - |
| Misiak et al. (2023a) | 18 | 2 (11.1) | - | - | - | - |
| Misiak et al. (2024) | 29 | 1 (3.4), 4 (13.8) | - | - | - | - |
| Misiak et al. (2023b) | 35 | 7 (20.0) | - | - | - | - |
| Nunez et al. (2018) | 15 | 1 (6.7) | 2 (13.3) | - | - | - |
| Nunez et al. (2020) | 22 | 6 (27.3) | - | - | - | - |
| Qiao et al. (2024) | 26 | 7 (26.9) | 13 (50.0) | 18 (69.2) | 7 (26.9) | - |
| Rejek and Misiak (2023) | 11 | 7 (63.6), 4 (36.4) | - | - | - | - |
| Scott et al. (2021) | 20 | 2 (10.0), 3 (15.0) | 3 (15.0), 1 (5.0) | 2 (10.0), 2 (10.0) | - | - |
| Suen et al. (2024) | 21 | - | - | - | 12 (57.1) | 11 (52.4) |
| Astill Wright et al. (2023) | 45 | 1 (2.2) | - | - | - | 5 (11.1) |
| Xavier et al. (2022) | 14 | 4 (28.6) | - | - | - | - |
| Yang et al. (2023) | 6 | 1 (16.7) | - | - | - | - |
| Zhou et al. (2023) | 22 | 12 (54.5) | - | - | 12 (54.5) | - |
| Overall | – | 23.5 | 26.0 | 29.7 | 26.9 | 29.1 |

Data expressed as the highest rank of PLEs in the network (percentage rank with respect to the total number of nodes in the network.

**Table 2.** A detailed summary of findings from included studies.

| Topic addressed | Study | Summary of findings |
| --- | --- | --- |
| Phemelogy of PLEs and associated symptom domains | Astill Wright et al. (2023) | Three clusters of symptoms were identified: PLEs; PTSD symptoms; and depressive and anxiety symptoms and negative symptoms of psychosis. PLEs had the strongest associations with other symptoms in the network. Symptoms of anxiety played a key role in bridging PLEs, PTSD symptoms, and depressive symptoms. |
|  | Cernis et al. (2021) | Dissociation was found to be highly connected to other des. It was directly connected to hallucinations, grandiosity, paranoia, cognitive disorganization, anxiety, depression, and PTSD symptoms. The directed graphs analysis, it was found that dissociation more likely influences paranoia, and cognitive disorganization than *vice versa*. Moreover, dissociation was found to be a probable influence of insomnia and distress tolerance through indirect pathways. |
|  | Cheng et al. (2024) | “Failure”, “External control”, and “Lack of activity” were found to be the most central des. The main bridge des between PLEs and depressive symptoms were “failure” (depressive symptoms), “guilty” (depressive symptoms), and “no future” (depressive symptoms). “Odd looks” (PLEs) and “unable to terminate” (negative symptoms) were the most relevant to the des of depressive symptoms. |
|  | Fung et al. (2024) | PTSD and dissociative symptoms were associated with higher levels of PLEs. Identity dissociation was particularly associated with perceptual anomalies and bizarre experiences, while emotional constriction was particularly related to negative symptoms. |
|  | Hajduk et al. (2023) | Four highly stable and densely connected communities within the network were identified including social relationships, autistic traits, positive symptoms, and the last one consisting of all negative symptoms, problems in social interactions, and depressive symptoms. The most central des in the network were difficulties in social interaction, perceived rejection, bizarre ideas, depression, and social withdrawal. |
|  | Misiak et al. (2023b) | Four pathways linking narcissistic grandiosity with PLEs, through the effect of one mediating de, were found. These mediating des included external attribution biases, the need to control thoughts, social cognition, and emotion regulation through fantasizing. The shortest pathway led through the effects of external attribution biases. Age, gender, education and lifetime history of psychiatric treatment were included as covariates in a network analysis. |
|  | Misiak and Frydecka (2024) | The intent to seek treatment was connected to five des of PLEs including “deja vu experiences”, “problems in differentiating reality and imagination”, “a lack of control over own ideas or thoughts”, “being distracted by distant sounds”, and “paranoid thoughts”. |
|  | Murphy et al. (2018) | The network analysis was limited to PLEs and their characteristics. The des of paranoia were the most central variables. The binary impairment network structure was similar to the occurrence network. However, the impairment network had significantly stronger interconnectivity of PLEs. |
|  | Rejek and Misiak (2023) | Two networks were analyzed (i.e., with symptoms operationalized using existing thresholds and as continuous variables). PLEs were directly linked with OCD symptoms, manic symptoms, depressive symptoms, and ADHD symptoms in both networks. Edge weight for the connection of PLEs and OCD symptoms was significantly higher compared to edge weights of all other direct connections of PLEs with psychopathology in both networks. |
|  | Scott et al. (2021) | The four most important symptoms that differentiated recent onset bipolar disorder from other cohort participants were: anergia, psychomotor speed, hypersomnia and loss of confidence. The four most important symptoms that differentiated individuals at high risk of bipolar disorder from unipolar depression (based on a family history of bipolar disorder) were anergia, psychomotor speed, impaired concentration, and hopelessness. Notably, PLEs were t found among differentiating clinical characteristics. |
|  | Suen et al. (2024) | Individuals with comorbid ASD and ADHD had significantly higher levels of PLEs. Baseline ASD symptoms (“heightened sensitivity to sensory input”) and ADHD symptoms (“difficulties in sustaining attention and task completion”) predicted the occurrence of PLEs at one-year follow-up. |
|  | Wusten et al. (2018) | PLEs showed a significantly higher network density in high-income countries compared to low- and middle-income countries. The level of associated distress was also higher in high-income countries. However, PLEs were significantly less frequently reported in high-income countries. The findings point to cultural differences with respect to endorsement of PLEs and associated distress. PLEs might have a greater relevance in high-income countries. |
|  | Xavier et al. (2022) | Discrepancies between youth and caregiver symptom reports were higher than average for Black youth and for youth representing low socioeconomic status. Discrepancies were also higher than average in youth with any psychiatric disorder when compared to typically developing youth. These differences were also found for PLEs. |
|  | Yang et al. (2023) | Domains of psychopathology associated with PLEs were analyzed. Both depressive and anxiety symptoms were most strongly associated with persecutory ideation while manic symptoms were most closely related to bizarre experiences. |
| Exposure to stress and PLEs | Betz et al. (2023) | Gender was the most important contributing factor to heterogeneity observed in symptom networks. However, heterogeneity was also explained by interpersonal trauma (childhood abuse and domestic violence) in women. Also, domestic violence, cannabis use, and ethnicity explained heterogeneity in men. In women, especially those with a history of early interpersonal trauma, affective symptoms in psychosis might be of importance. Men, especially those from minority ethnic groups, showed a strong network connection between hallucination-like experiences and persecutory ideation. |
|  | Deng et al. (2023) | Interpretation inflexibility was associated with social functioning impairment. Affective symptoms and paranoia mediated the association. These associations were magnified by stress experienced during the COVID-19 pandemic, i.e., a moderated mediation was found only in relation to affective symptoms but not paranoia. A network analysis confirmed the moderating effects of the COVID-related preoccupation on the association between interpretation inflexibility and depression. |
|  | Gaweda et al. (2021) | Various categories of childhood trauma were the most central des in the network. The association of childhood trauma history with PLEs was found to be mediated by depressive symptoms and cognitive biases. However, the shortest pathways led through various categories of childhood trauma. |
|  | Huang et al. (2023) | A history of childhood trauma was closely connected to schizotypy and motivation. Compared to the low schizotypy subgroup, the network of the high schizotypy subgroup showed higher global strength. The two subgroups did t differ in network structure. Network analysis using the replication dataset revealed similar findings. |
|  | Qiao et al. (2024) | The symptoms of general psychopathology (i.e., anxiety, hostility, and somatization) were the main connecting components between childhood trauma history and PLEs. Longitudinal analysis in a subsample with wave 2 data demonstrated that variables with the highest centrality (i.e., depression, negative affect, and loneliness) predicted follow-up PLEs. |
|  | Rejek and Misiak (2024) | A network analysis revealed that the exposome score (a cumulative measure of environmental exposures) was directly connected not only to items representing psychotic experiences (“paranoid thoughts”, “a lack of control over own ideas or thoughts”, “thought echo”, and “being distracted by distant sounds”) but also to those representing depressive or anxiety symptoms (“uninterested in things used to enjoy” and “feeling anxious when meeting people for the first time”). |
|  | Sun and Zhong (2023) | Four dimensions of perceptual abnormalities were identified: aberrant bodily perceptions, altered daily experiences, chemosensation (i.e., abnormal gustatory and olfactory experiences), and clinical psychosis (i.e., visual and auditory hallucinatory experiences). Physical bullying and cyberbullying were directly and positively linked to two of the aberrant bodily experiences. |
| PLEs and suicide-related outcomes | Fonseca-Pedrero et al. (2021) | The occurrence of PLEs was related to higher levels of suicidal ideation and behaviors, depressive symptoms as well as emotional and behavioral difficulties. |
|  | Misiak et al. (2024) | PLEs were connected to the de of current suicidal ideation only in participants with a higher severity of insomnia. The des of PLEs connected to the current suicidal ideation de were deja vu experiences, auditory hallucination-like experiences and paranoia. |
|  | Misiak et al. (2023c) | A history of childhood sexual abuse was the only category of childhood trauma that was directly related to the characteristics of NSSI (i.e., longer lifetime duration of NSSI). The shortest pathways from other categories of childhood trauma led to the lifetime characteristics of NSSI through a history of sexual abuse. Other pathways were also possible and converged on des representing persecutory thoughts, déjàvu experiences, psychomotor retardation/agitation and suicidal ideation. These symptoms were the only des directly connected to the characteristics of NSSI (i.e., lifetime duration and a history of severe NSSI). |
|  | Nunez et al. (2018) | Lifetime suicidal ideation was mostly connected to the PLEs represented by perceptual anomalies and bizarre experiences. |
|  | Nunez et al. (2020) | Lifetime suicidal ideation was directly connected to nodes representing bizarre experiences, perceptual abnormalities, social anxiety, depressive symptoms, and negative symptoms. The de “I felt I had thing to live for” had the strongest connection to lifetime suicidal ideation. |
|  | Zhou et al. (2023) | The severity of depressive symptoms was the only factor connected to both NSSI and suicide. Other variables, including PLEs, were linked through the bridging effect of depressive symptoms. |

**References**

Astill Wright, L., McElroy, E., Barawi, K., Roberts, N.P., Simon, N., Zammit, S., Bisson, J.I., 2023. Associations among psychosis, mood, anxiety, and posttraumatic stress symptoms: A network analysis. J Trauma Stress 36, 385-396.

Betz, L.T., Penzel, N., Rosen, M., Bhui, K., Upthegrove, R., Kambeitz, J., 2023. Disentangling heterogeneity of psychosis expression in the general population: sex-specific moderation effects of environmental risk factors on symptom networks. Psychol Med 53, 1860-1869.

Cernis, E., Evans, R., Ehlers, A., Freeman, D., 2021. Dissociation in relation to other mental health conditions: An exploration using network analysis. J Psychiatr Res 136, 460-467.

Cheng, P., Liu, Z., Sun, M., Zhang, W., Guo, R., Hu, A., Long, Y., 2024. The relations of psychotic-like experiences (PLEs) and depressive symptoms and the bias of depressive symptoms during the clustering among Chinese adolescents: Findings from the network perspective. J Affect Disord 350, 867-876.

Deng, W., Everaert, J., Bronstein, M.V., Joormann, J., Cann, T., 2023. Social Interpretation Inflexibility and Functioning: Associations with Symptoms and Stress. J Soc Clin Psychol 42, 29-49.

Fonseca-Pedrero, E., Muniz, J., Gacia-Portilla, M.P., Bobes, J., 2021. Network structure of psychotic-like experiences in adolescents: Links with risk and protective factors. Early Interv Psychiatry 15, 595-605.

Fung, H.W., Wong, M.Y.C., Moskowitz, A., Chien, W.T., Hung, S.L., Lam, S.K.K., 2024. Association Between Psychotic and Dissociative Symptoms: Further Investigation Using Network Analysis. J Trauma Dissociation 25, 279-296.

Gaweda, L., Pionke, R., Hartmann, J., Nelson, B., Cechnicki, A., Frydecka, D., 2021. Toward a Complex Network of Risks for Psychosis: Combining Trauma, Cognitive Biases, Depression, and Psychotic-like Experiences on a Large Sample of Young Adults. Schizophr Bull 47, 395-404.

Hajduk, M., Strakova, A., Januska, J., Ivancik, V., Dancik, D., Cavojska, N., Valkucakova, V., Heretik, A., Pecenak, J., Abplanalp, S.J., Green, M.F., 2023. Connections between and within extended psychosis and autistic phetypes and social relationships in the general population. J Psychiatr Res 157, 36-42.

Huang, Y.H., Hu, H.X., Wang, L.L., Zhang, Y.J., Wang, X., Wang, Y., Wang, Y., Wang, Y.Y., Lui, S.S.Y., Chan, R.C.K., 2023. Relationships between childhood trauma and dimensional schizotypy: A network analysis and replication. Asian J Psychiatr 85, 103598.

Misiak, B., Frydecka, D., 2024. Psychotic-like experiences predict the perceived intent to seek treatment: A network perspective. Schizophr Res 266, 100-106.

Misiak, B., Gaweda, L., Moustafa, A.A., Samochowiec, J., 2024. Insomnia moderates the association between psychotic-like experiences and suicidal ideation in a n-clinical population: a network analysis. Eur Arch Psychiatry Clin Neurosci 274, 255-263.

Misiak, B., Kowalski, K., Jaworski, A., Swirkosz, G., Szyszka, M., Piotrowski, P., 2023a. Understanding pathways from narcissistic grandiosity to psychotic-like experiences: Insights from the network analysis. J Psychiatr Res 166, 122-129.

Misiak, B., Szewczuk-Boguslawska, M., Samochowiec, J., Moustafa, A.A., Gawęda, Ł., 2023b. Unraveling the complexity of associations between a history of childhood trauma, psychotic-like experiences, depression and n-suicidal self-injury: A network analysis. J Affect Disord.

Murphy, J., McBride, O., Fried, E., Shevlin, M., 2018. Distress, Impairment and the Extended Psychosis Phetype: A Network Analysis of Psychotic Experiences in an US General Population Sample. Schizophr Bull 44, 768-777.

Nunez, D., Fres, A., van Borkulo, C.D., Courtet, P., Arias, V., Garrido, V., Wigman, J.T.W., 2018. Examining relationships between psychotic experiences and suicidal ideation in adolescents using a network approach. Schizophr Res 201, 54-61.

Nunez, D., Monjes, P., Campos, S., Wigman, J.T.W., 2020. Evidence for Specific Associations Between Depressive Symptoms, Psychotic Experiences, and Suicidal Ideation in Chilean Adolescents From the General Population. Front Psychiatry 11, 552343.

Qiao, Z., Lafit, G., Lecei, A., Achterhof, R., Kirtley, O.J., Hiekkaranta, A.P., Hagemann, N., Hermans, K., Boets, B., Reininghaus, U., Myin-Germeys, I., van Winkel, R., 2024. Childhood Adversity and Emerging Psychotic Experiences: A Network Perspective. Schizophr Bull 50, 47-58.

Rejek, M., Misiak, B., 2023. Dimensions of psychopathology associated with psychotic-like experiences: Findings from the network analysis in a nclinical sample. Eur Psychiatry 66, e56.

Rejek, M., Misiak, B., 2024. Modelling the effects of the exposome score within the extended psychosis phetype. J Psychiatr Res 169, 22-30.

Scott, J., Crouse, J.J., Ho, N., Carpenter, J., Martin, N., Medland, S., Parker, R., Byrne, E., Couvy-Duchesne, B., Mitchell, B., Merikangas, K., Gillespie, N.A., Hickie, I., 2021. Can network analysis of self-reported psychopathology shed light on the core phemelogy of bipolar disorders in adolescents and young adults? Bipolar Disord 23, 584-594.

Suen, Y.N., Chau, A.P.Y., Wong, S.M.Y., Hui, C.L.M., Chan, S.K.W., Lee, E.H.M., Wong, M.T.H., Chen, E.Y.H., 2024. Comorbidity of autism spectrum and attention deficit/hyperactivity disorder symptoms and their associations with 1-year mental health outcomes in adolescents and young adults. Psychiatry Res 331, 115657.

Sun, X., Zhong, J., 2023. The dimensionality of perceptual amalies and their relationships with bullying victimization among Chinese adolescents: From a network perspective. Schizophr Res 262, 42-50.

Wüsten, C., Schlier, B., Jaya, E.S.; Genetic Risk and Outcome of Psychosis (GROUP) Investigators; Fonseca-Pedrero, E., Peters, E., Verdoux, H., Woodward, T.S., Ziermans, T.B., Lincoln, T.M., 2018. Psychotic Experiences and Related Distress: A Cross-national Comparison and Network Analysis Based on 7141 Participants From 13 Countries. Schizophr Bull 44, 1185-1194.

Xavier, R.M., Calkins, M.E., Bassett, D.S., Moore, T.M., George, W.T., Taylor, J.H., Gur, R.E., 2022. Characterizing Youth-Caregiver Concordance and Discrepancies in Psychopathology Symptoms in a US Community Sample. Issues Ment Health Nurs 43, 1004-1013.

Yang, X.H., Zhang, J.W., Li, Y., Zhou, L., Sun, M., 2023. Psychotic-like experiences as a co-occurring psychopathological indicator of multi-dimensional affective symptoms: Findings from a cross-sectional survey among college students. J Affect Disord 323, 33-39.

Zhou, H.Y., Luo, Y.H., Shi, L.J., Gong, J., 2023. Exploring psychological and psychosocial correlates of n-suicidal self-injury and suicide in college students using network analysis. J Affect Disord 336, 120-125.
